# Medical Wards of Mercy Hospital

**Published:** 1872-03-01

**Authors:** N. S. Davis


### MEDICAL WARDS OF MERCY HOSPITAL.

SERVICE OF PROF. N. S. DAVIS.—[*Reported by B.*]

CASE I. ACUTE RHEUMATISM—RHEUMATIC FEVER.—This patient is a middle-aged man, laborer, of fair physical development, and until recently enjoying good health. He was admitted to the hospital ward two days since, nearly two weeks after the commencement of his sickness. His face is flushed and expressive of suffering; his skin hot, and covered with profuse perspiration; respiration more frequent than natural; pulse full, firm, and 100 per minute; tongue covered with a white coat; bowels torpid; urine scanty and high-colored; the wrists, ankles, knees, and hips moderately swollen and very painful, the pain greatly aggravated by motion; the heart's sounds are distant, but without any rough or valvular murmur, and the apex does not apparently strike the walls of the chest, indicating the probability of some pericardial effusion. This is a plain case of acute rheumatism, of considerable severity.

It was remarked that in the progress of such cases the cardiac structures were extremely liable to become involved in the inflammation, which should cause the practitioner carefully to note the cardiac sounds often, while attending this class of patients. To correct the excessive acidity of the fluids, which exists in nearly all cases of acute rheumatism, to diminish the plastic exudations into the inflamed fibrous tissues surrounding the joints, and to mitigate the constant suffering of the patient, he was directed on admission to take one tablespoonful of the following solution every three hours:

| | | |
|---|---|---|
| ℞ | Bi-Tart. Potassa | ʒ iii. |
| | Sulph. Morph. | 2 grs. |
| | Water | ℥ viii. |

Mix.

Also a powder of Pulv. Doveri, 8 grs., and Calomel, 1 gr., each night and morning. On account of the profuse sweating, which is rather encouraged by the bi-tartrate of potassa, the following mixture was given as a substitute:

℞ Tinct. Cimicifuga Rac ℥ ii.

Vin. Colchici ℥ i.

Camp. Tinct. Opii ℥ i.

Tinct. Verat. Viride ʒ i.

Nit. Potassa ʒ ii.

Mix, and take one teaspoonful every three hours in sweetened water; and continue one dose of the pulv. doveri and calomel at bedtime. The lecturer stated that he had used the cimicifuga in the treatment of acute and sub-acute rheumatism for many years, and in most instances with benefit.

Note.—On visiting the ward one week after the foregoing clinic, the patient was convalescent. The only change in the treatment had been the omission of the calomel at night.

Case II. Rheumatic Bronchitis with Renal Congestion.—This man, a sailor, aged about 25 years, was admitted into the Hospital one week since. At the time his face was turgid, and some bloating over the whole body of a semi-œdematous character, with a look of spanæmia. His breathing was short and hurried; pulse quick and irritable; paroxysms of severe, harsh cough; expectoration scanty, but sometimes mixed with streaks of blood; respiratory murmur harsh and exaggerated, with some dry rhonci, but no marked dullness or percussion. There was also well marked rheumatic pains in the joints of the lower extremities, and the urine was scanty and high colored. Previous to the attack the patient had been exposed to cold and wet on the lake, and the case was regarded as rheumatic inflammation of the bronchial tubes; especially of the smaller tubes, with congestion of some lobules of the left lung. It was remarked that rheumatic bronchitis was not unfrequent in our climate, and was generally characterized by harshness and severity of cough, scantiness of expectoration, constriction or tightness in the chest, sometimes amounting to asthmatic breathing, especially at night, and the coincidence of rheumatic pains and swellings in other parts.

To correct the rheumatic diathesis, and mitigate the severity of the cough, this patient was given 10 grs. of bi-tartrate of potassa and 10 grs. of Dover's powder, every four hours, and a teaspoonful of the following mixture between:

℞ Hydrochlorate of Ammonia ʒ iii.

Tart. Ant. et Pot. grs. ii.

Sulph. Morph. grs. iii.

Vin. Colchici ℥ ss.

Syrup Liquorice ℥ iiiss.

Mix.

This treatment has now been continued one week, and all the symptoms are much relieved. The soreness, cough and sense of tightness in chest are so much relieved that the patient rests comfortably at night, and the urine is much more abundant. He was directed to continue the hydrochlorate of ammonia mixture before each meal time, and the Dover's powder and bi-tartrate only at bed-time.

Case III. Pleurisy with Effusion.—This is a young man, admitted into the Hospital two days since. His face was deeply suffused with a purplish flush; lips pale; extremities cool, but surface of trunk hot and dry; pulse 120 per minute, moderately firm; respirations short and 30 per minute, but no cough, no expectoration, and no pain in the chest; bowels inactive, and urine scanty. He had been sick about one week before admission. Inspection of the chest showed flattening of the left infra-clavicular region, with slight dullness, and tubular respiration; the right side was less flattened antero-posteriorly, but very dull on percussion over the whole lower and lateral part, with silence on auscultation. These symptoms and signs indicated latent pleurisy with effusion on the right side, with tuberculosis of the upper lobe of the left lung. His treatment had been moderately sedative and diuretic, consisting of one teaspoonful of the following mixture every four hours:

℞ Comp. Syrup Squills ℥ ii.

Camph. Tinct. Opii ℥ ii.

Tinct. Verat. Viride ʒ i.

Mix.

Also, between each of the above doses, a fluid drachm of a mixture of equal parts of liquor ammonia acetatis and nitrous ether. This treatment has now been followed two days, and the veratrum has rendered the pulse slower and more soft, with less suffusion of the face, and less heat of surface over the chest.

On account of these changes, and a slight feeling of nausea, the veratrum was directed to be omitted from the expectorant and anodyne mixture, and twenty drops of tincture of digitalis to be added to each dose of the liquor ammonia acetatis and nitrous ether. Also more nutritious diet.

SERVICE OF PROF. D. T. NELSON.

CASE IV. GOITRE—ENLARGEMENT OF THYROID GLAND.—This patient has a moderate enlargement of the thyroid gland, presenting a prominence across the lower front part of the neck. It was first noticed by the patient six weeks since, and has been slowly increasing until she applied for treatment last week. The swelling is neither painful nor tender, and is chiefly interesting on account of its liability to increase until its prominence becomes unseemly and more or less oppressive to the trachea. In our country the disease is comparatively rare, while in some parts of Europe it is very common. She has been taking small doses of iodine in solution with iodide of potassa, and there is already a decided diminution in the size of the swelling. The same treatment was continued.

CASE V. AMENORRHŒA—ACNÆ.—This is a young woman who has failed to menstruate at the proper age, and has become decidedly anæmic. She has also become subject to regular daily attacks of severe pain in the epigastrium; the pain coming at the same hour each day.

For a tonic she has been taking the syrup of pyrophosphate of iron, a teaspoonful three times a day. Sometimes this was superseded by the following tonic and laxative pills:

| | | |
|---|---|---|
| ℞ | Pulv. Aloes | 30 grs. |
| | Sulph. Ferri | 45 grs. |
| | Ext. Belladonna | 5 grs. |
| | Strychnia | 1 gr. |

Mix. Divide into 30 pills, take 1 before each meal time.

To interrupt the daily paroxysms of gastric pain she was given sulphate of quinia each day, a little before the expected paroxysm, which entirely relieved this symptom. She was also troubled with pustules of Acnæ on her face, which have been much improved by the simple application of glycerine daily as a wash.

SERVICE OF LYMAN WARE, M. D.

CASE VI. This woman has syphilitic ulcers in the nostrils, and presents the odor of ozena intensified by the fetor of necrosis. The septum of the nose is partially destroyed by the ulcerative process. She had been two years without the specific treatment, which should have been adopted at once.

In all such cases, and even in such as present a somewhat doubtful diagnosis, the early and judicious use of mercurials, followed by the iodide of potassa, is of great importance. When the disease has advanced to the stage presented in this case, the following constitutes one of the most efficient combinations that can be given internally:

| | | |
|---|---|---|
| ℞ | Iodide Soda | ʒ iii. |
| | Bi-chlorid Hydrag. | gr. i. |
| | Fl. Ext. Conium | ℥ ss. |
| | Syrup Prunus Virg. | ℥ iss. |
| | Aqua | ℥ ii. |

Mix, and give one teaspoonful before breakfast, dinner and bed time. Besides the specific, internal medication, local remedies are required by the condition of the nostrils. The parts should be thoroughly cleansed by using suitable washes with the nasal douche or the nasal syringe. A simple solution of sugar in warm water, used daily, makes a mild, cleansing wash. In this case diluted tincture of iron will be used with the atomizer twice per week. To destroy the offensive odor the patient may have a powder composed of sub-nitrate of bismuth ʒ v. and chlorate of potassa ʒ i., to be used as a snuff several times a day. In syphilitic cases it is better to put calomel with the bismuth, instead of the chlorate of potassa, for a few days at a time, care being taken not to have it used long enough at one time to endanger the absorption of sufficient mercurial to produce salivation.

---

THE steam from a mixture of an ounce of aqua ammonia to a quart of water, inhaled once a day, is said by Mr. John Grantham, *Brit. Med. and Surg. Journal*, to relieve and even cure attacks of whooping-cough.

